# Children’s Failure in Analogical Reasoning Tasks: A Problem of Focus of Attention and Information Integration?

**DOI:** 10.3389/fpsyg.2017.00707

**Published:** 2017-05-23

**Authors:** Yannick Glady, Robert M. French, Jean-Pierre Thibaut

**Affiliations:** LEAD – CNRS UMR 5022, Université de Bourgogne Franche-Comté, Dijon, France

**Keywords:** analogy, analogical reasoning, cognitive development, task organization, processing constraints, information availability

## Abstract

Children’s improved performance with age in analogy tasks has been explained by an increase in semantic knowledge of the items and the relations between them or by the development of an increased ability to inhibit irrelevant information. We tested the so-called “unbalanced attentional focus hypothesis” that claims that a failure to choose the “analogical” match can be the result of a difficulty to focus on all the relevant information available. Previous eye-tracking research has suggested, in analogies of the A:B::C:D format, that 5–6 year-olds organize their search around the C item. They focused significantly less than adults on the A:B pair, thereby hindering their discovering the relation(s) between A and B. We hypothesized that inducing them to focus their attention on the A:B pair at the beginning of the trial would affect their performance. In Experiment 1, increasing children’s focus on the A:B pair did, indeed, lead to better performance. In contrast, in Experiment 2, focusing their attention on the A:B pair impaired performance when the most salient relation holding between A and B was, in fact, irrelevant for the analogy. By contrast, the obvious-but-irrelevant relation in the A:B pair had no negative effect on performance when no explicit A:B focusing was induced. These results are discussed in terms of the temporal organization of the task and availability of information, and of children’s difficulties to disengage from the main goal of the task, when necessary.

## Introduction

Analogy making is a fundamental process in everyday problem solving, as well as in refined human activities like art and creation, argumentation, and science (Holyoak, 2012; Hofstadter and Sander, 2013) and plays a key role in conceptual development (e.g., Gentner, 2010). It refers to the process of comparison between the representations of a source and a target domain, in terms of common relations between the items composing these two domains, despite important differences between the elements to be compared. For example, one can make an analogy between sound waves and water waves. Or, the same “part of” relation can be drawn between arm-body and wheel-car, i.e., in two quite different conceptual domains. Analogies are also used in problem solving in many domains (e.g., mathematics, science, law), when a known solution in one semantic field is applied to another field (Gick and Holyoak, 1980). All of these situations require finding the common relation(s) holding in both domains, and have been studied for different tasks, such as comprehension, construction, generation, problem solving tasks (see Holyoak, 2012).

Mapping is the hallmark of analogy (Gentner, 1983; Holyoak, 2012). It is a comparison process that involves the alignment of elements from both domains (Markman and Gentner, 1993) and the generation of inferences between the base and the target domains. Consider, for example, the analogy “bird is to nest as dog is to? (solution: *doghouse*),” written as *bird:nest::dog:? (doghouse)* in the standard A:B::C:? framework (also called “proportional analogy”). Analyzing the A:B pair (i.e., *bird* and *nest*) produces a relation (or relations) between these two items (i.e., here “lives in”) that can be applied to the target domain. Here, *bird* will be aligned (i.e., put into correspondence) with *dog* and *nest* with *doghouse*. Analogical reasoning requires building a representation of the A–B and C–D pairs and retrieving information associated with the items making up the pairs from memory. Mapping the pairs will then involve comparisons both within and between the items in the base and target pairs in order to find a common relational system (i.e., that can be applied to both domains) (see French, 2002; Gentner and Forbus, 2011; Holyoak, 2012). When a unifying relation between the base pair and possible target pairs is difficult to find, more comparisons must take place. This often requires re-representation of (one of) the pair(s) in order to improve the overall relational match between base and target pairs (e.g., Kokinov et al., 2007). This re-representation frequently entails finding novel relations unifying the pairs. By definition, to produce a valid analogy, the final alignment must be between items that constitute relationally consistent pairs (Gentner, 1983). This means that the same relation holds within each pair and that equivalent terms in the two pairs (e.g., bird and dog) are found. The A:B::C:D format has been widely used in the developmental literature (e.g., Goswami and Brown, 1990; Rattermann and Gentner, 1998; Gentner, 2010; Thibaut et al., 2010a,b, among many others). In scene analogies, two scenes are introduced in which there is an interaction between characters or objects or between characters and objects (e.g., a dog is chasing a cat in one scene and a boy is chasing a girl in the other scene). The experimenter points to an entity in one scene (e.g., the dog) and participants are asked to find the entity that plays the same role in the other scene (e.g., the boy), which requires, first, identifying the relation and, second, identifying the role (see Markman and Gentner, 1993; Richland et al., 2006). In any case, solving semantic analogies depends on semantic associations. Indeed, even in young children, semantic relations influence children’s processing of words by the end of the second year of age (Arias-Trejo and Plunkett, 2009). Semantic relatedness is known to influence young children’s processing of semantic analogies. For example, Thibaut et al. (2010a) studied the role of the semantic association strength between items making up the A–B and C–D pairs with 4- and 5-year-old children. They compared weak and strong analogies (i.e., analogies in which the items making up the A–B and C–D pairs were either weakly or strongly associated, e.g., “dress” and “hanger” are weakly related whereas “bee” and “hive” are strongly related according to adults’ judgments) and manipulated the number of semantic distractors (1 or 3) present in the set of possible solutions. Their results revealed a difference between weak and strong analogies, only with three distractor items. Moreover, strong analogies were largely unaffected by the number of distractors. This was probably due to the fact that the relations between the A–B and C–D item pairs were sufficiently strong that they were not interfered with by the semantic distractors. In contrast, when the problem involves weakly associated items, mapping the A–B pair onto the C–D pair requires more than simply accessing an obvious, shared semantic relation between the A–B and C–D items and the problem is, therefore, more difficult to solve (see also Arias-Trejo and Plunkett, 2009).

The present paper highlights a number of factors that might influence children’s search for a solution. Our central hypothesis, which we call the *unbalanced attentional focus hypothesis*, is that *young children fail to solve analogies because they have difficulty focusing on and integrating all the information available in the problem* (Thibaut et al., 2011a; Thibaut and French, 2016). We hypothesized that manipulating the amount of attention toward information that children generally pay less attention to than adults would impact their performance.

### The Development of Analogical Reasoning: Theories

Analogical reasoning has given rise to a large body of developmental data, including data from aging people and people with neurodegenerative diseases (e.g., Viskontas et al., 2004; Bugaiska and Thibaut, 2015). These data have been generated from various paradigms built around the classical A:B::C:D analogies (e.g., Goswami and Brown, 1990; Thibaut et al., 2010a,b), scene analogies (e.g., Richland et al., 2006, see also Markman and Gentner, 1993), analogical problem solving (Holyoak et al., 1984), or metaphors (Gentner, 1988). Across ages, it has been shown that school-aged children use analogies to enhance their understanding of concepts in biology (e.g., Brown and Kane, 1988) and physics (e.g., Pauen and Wilkening, 1997). It has also been shown that young infants can reason spontaneously by analogy to solve problems (around 18 months in Chen et al., 1997; or 3-to-4 year olds, Goswami and Brown, 1990; Tunteler and Resing, 2002). One way to conceptualize children’s development of analogical reasoning is to say that children undergo a ‘relational shift’ (Rattermann and Gentner, 1998). In this framework, analogical reasoning for younger children would initially be based on the surface features of stimuli (e.g., shape, color, texture as shown by same-shape or same-color lures) and would later include information about the relations between entities, ultimately incorporating complex systems of relations.

This progression is explained either by the accretion of relational knowledge or by the maturation of executive functions (EFs), i.e., including working memory, inhibition or flexibility. A brief overview of these two currents follows.

(1) Knowledge accretion favors relational reasoning

This view posits that one’s performance on analogical reasoning tasks can be explained in terms of a gradual increase of his/her structured knowledge of the world (Goswami and Brown, 1990; Goswami, 1991, 2001). According to Goswami and Brown (1990), children are able to map relations from early infancy, as long as they have the necessary relational knowledge. According to Goswami and Brown (1990) and Rattermann and Gentner (1998), the ability to make relational comparisons in one domain increases with the accretion of relational knowledge in the corresponding domain. Note that this explanation does not refer to the cognitive costs associated with gaining more knowledge. Interactions between knowledge accretion and cognitive costs have been discussed by Richland et al. (2006) or Thibaut et al. (2010a). Goswami and Brown (1990) have argued that children’s failures with analogies used in earlier research by Piaget et al. (1977), such as bicycle:handlebars::ship:? (answer: rudder) could not be solved by children simply because they did not know that rudders steer ships. These vocabulary deficiencies can be revealed by appropriate testing and are sufficient to explain failures to solve analogy problems involving these words/concepts (see Richland et al., 2006; Thibaut et al., 2010a for discussions).

(2) Executive functions (EFs)

Other authors have proposed that the maturation of EFs is involved in the development of analogy-making skills (Halford et al., 1998; Waltz et al., 2000; Richland et al., 2006; Thibaut et al., 2010b; Morrison et al., 2011). Components of EFs such as inhibition and cognitive flexibility (Anderson, 2002; Diamond, 2013) are involved in analogical reasoning. Analogical reasoning requires selecting the relational information that is relevant to the analogy, which might require testing several relations and rejecting irrelevant information (e.g., semantic and/or perceptual distractors). For example, if A and B are, respectively, a *bird* and a *nest*, and C is a *dog*, then D should be a *doghouse*. Highly semantically-related-to-C distractors, such as *bones* or *cat*, must be actively inhibited as solutions to the analogy. Richland et al. (2006), Thibaut et al. (2010a), Morrison et al. (2011) stress the importance of children’s ability to “inhibit tendencies to respond on the basis of competing superficial similarities” (Rattermann and Gentner, 1998; Richland et al., 2006, p. 253). Thibaut et al. (2011b) showed that in 5-year-old children inhibition capacities correlated with performance in an A:B::C:D task (see Morrison et al., 2004, with adult patients), or that the number of errors increase with the number of distractors, even though children knew the analogical relation, as shown by an independent control (Thibaut et al., 2010a). In short, investigations connecting the development of analogical reasoning and EFs have mainly focused on the different sources of information (e.g., featural or relational similarities, number and types of distractors). As far as we know, no study to date on analogical reasoning has focused on the way information is made available during the task and on the effect of explicitly asking young children to focus on the base domain (here, to verbalize the A–B relation). This is the main goal of the present paper.

### Inhibition, Flexibility, and Pacing the Analogy Task: Manipulating Information Availability and Naming the A:B Relation

The previous section examined the role of two general classes of explanations. Here we consider the structure of an analogy task, its requirements, and how these requirements might contribute to children’s difficulties with the task. Analogies involve multiple comparisons within and between the base and the target pairs that must be integrated (see French, 2002; Gentner and Forbus, 2011; Holyoak, 2012) and we claim that children’s failure to appropriately perform comparisons between the base and the possible-target pairs is a source of errors. The explicit goal of the task is “to find an analogical solution, the D, that goes with C in the same way as A goes with B”. However, finding a relationally consistent analogical solution first requires an analysis of the base pair (i.e., the A:B pair) in order to find potential relations unifying A and B that can then be applied to the target pair. By using an eye-tracker to record children and adults’ gazes while they solved A:B::C:? problems, Thibaut et al. (2011a) and Thibaut and French (2016) showed that, unlike adults, children organize their search around the C term in the target pair from the outset, focusing less on the A:B pair than adults, even when they ultimately gave the correct answer to the problem. The authors hypothesized that children had difficulties temporarily inhibiting the main goal of the task (i.e., “to find an item, D, in a set of possible solutions that goes with C,” hereafter: “the C:?- main goal”), in order to focus on the subgoal of finding a relevant relation between A and B (henceforth: “A:B-subgoal”). In terms of EFs, finding a D that goes with C (i.e., the main goal of the task, the C:?- main goal), requires that one temporarily inhibits the C:?- main goal (while still keeping it in working memory), in order to study the A:B pair. This ability also involves cognitive flexibility because if participants spontaneously start with C, they will eventually have to shift toward the A:B pair to understand the analogy. Or, if they start with a relation holding between A and B that makes no sense for C and any item in the solution set, they will have to re-represent the relation holding in the A–B pair. Both inhibition and cognitive flexibility are under-developed in young children (Anderson, 2002).

If children do not spontaneously study the A:B pair, increasing their attention to it should help them to focus on it. This could be done by explicitly asking them to verbalize the relation. Indeed, we hypothesized that children’s verbalization of the relationship between A and B could contribute to an improved organization of their search for a solution, something that has not been considered in the analogy literature. We capitalize on the idea that language positively contributes to children’s performance (see Cragg and Nation, 2010, for review and Gruber and Goschke, 2004). For example, Kray et al. (2008) found a significant beneficial effect of task-relevant verbalization, especially for younger children and aging persons, two groups who did not spontaneously use this strategy. On the other hand, task-irrelevant verbalization interfered with the task. Similarly, in a dimension-switching task, Kirkham et al. (2003) showed that children’s performance was better when they had to verbalize the relevant dimension at the beginning of each trial (rather than having the experimenter label the dimension). In a similar vein, we asked children to explicitly verbalize the relation between A and B.

It has been argued that language contributes to analogical reasoning as a representation tool (e.g., Christie and Gentner, 2012). The representational role of language has been documented in situations in which children are provided with words (e.g., object or relation names) by contrast with a “no-word” condition (e.g., Loewenstein and Gentner, 2005). According to Christie and Gentner (2012), these results suggest that language plays what the authors call a *reifying* role while children are searching for correspondences between domains. Shared names encourage children to find items’ essential characteristics or deep relations connecting them (Gentner, 2010). As mentioned above, language would contribute to focusing on dimensions that would *a priori* be neglected, or at least would be less focused on than expected, if one wants to solve the task. As Wolff and Holmes (2011) put it, switching between dimensions improves when language contributes to highlighting conflicting dimensions (here, dimensions of the task, such the stimulus C and the set of solutions, on the one hand, and the A–B pair on the other).

### Goals of the Present Paper

In the present paper, we manipulated the temporal availability of information and the instructions given to the children before they started to perform the task (Klahr, 1985). These manipulations were supposed to influence the way children would temporally focus on the information while doing the task. We tested the hypothesis that children might fail because they do not optimally distribute their attention to the relevant components of the task. We call this the *unbalanced attentional focus hypothesis*. This hypothesis predicts that enhancing children’s attention toward part(s) of the analogy, specifically the A:B pair would influence their performance.

The present experiments manipulated two factors designed to increase the children’s initial focus on A and B. We then determined how each of these factors influenced analogical reasoning performance. The first factor, the temporal organization of the task, refers to the way the task components are introduced. The second, the verbalization of the A:B relation, refers to the request to verbalize (i.e., explicitly state) the relation between A and B. Because young children arguably have difficulties in spontaneously inhibiting the C:?- main goal, we “assist” their EFs by inducing them explicitly to focus on A:B. We highlight the A:B-subgoal by manipulating the moment of presentation of the A:B pair, presenting it before the other stimuli. In addition, we looked at the effect of asking participants to name the relation holding between A and B. Asking participants to name the relation in the A:B pair was intended to help them focus their attention on this pair and process it, something they naturally do less spontaneously than adults. These two manipulations were expected to focus children’s attention on the A:B pair, thereby contributing to a better integration of the A:B information required to solve the task. In the case of adults, Grant and Spivey (2003) showed, in Duncker’s radiation problem (Duncker, 1945), that more participants solved the problem when critical information was highlighted in comparison to control groups with no highlighting or with highlighting of non-critical information. In short, in contrast with previous studies, we have kept the analogies identical across conditions and (i) manipulated the way in which information was introduced (all the items composing a trial being introduced simultaneously vs. the A:B pair being introduced before the other items) and (ii) whether or not participants verbalized the relation between A and B.

In order to achieve these goals, in Experiment 1, we crossed the language factor (verbalization of the relation between A and B vs. no verbalization) with the type of presentation of the A:B pair (prior presentation of the A:B pair vs. simultaneous presentation of A:B along with all the other items). This resulted in four conditions in the A:B::C:? task, namely, (a) Standard (entire set of pictures simultaneously), (b) Standard+Verbalization (Standard plus being asked to verbalize the A:B relation), (c) A:B-first + No Verbalization (A:B shown before the other pictures, but no verbalization requested), (d) A:B-first + Verbalization condition (A:B shown before the other pictures + verbalization requested). We constructed the A:B pair in such a way that the obvious relation between A and B was the relation that gave the correct “analogical” answer when applied to C.

If children’s failures in analogy tasks resulted from over-focusing on the C:?-goal, at the expense of the A:B pair, we predicted that children should perform better in the (b), (c), and (d) conditions than in the (a) condition (i.e., the Standard condition) because verbalizing the relations between A and B and/or seeing the A:B pair first should contribute to greater focus on A:B.

In Experiment 2 we wished to determine if inducing a focus of attention on an obvious-but-irrelevant relation between A:B (“having the same color”) would interfere with children’s performance on solving an analogy problem that was not based on this irrelevant relation. For this, we compared the A:B-first + Verbalization condition to a slightly modified version of the Standard condition that was used in Experiment 1. We predicted that the irrelevant relation (i.e., same color) would interfere more with analogical reasoning in the A:B-first + Verbalization condition than in the modified version of the Standard condition.

## Experiment 1

Given that children spend less time than adults on the A:B pair (Thibaut et al., 2011a), the first experiment was designed to assess the role of language (asking participants to verbalize the relation holding between A and B vs. not verbalizing it) on children’s ability to solve analogy problems. We also manipulated when the A:B pair was shown, i.e., either before the presentation of C and the solution set, or at the same time as all of the other items making up the problem. These two factors were crossed resulting in a between-participants design with four experimental conditions. As in a number of previous studies, including our own, we chose 4-to-6-year olds because they are old enough to understand the task, they knew the stimuli composing the analogies, but do not yet have fully developed EFs (e.g., Richland et al., 2006).

### Methods

#### Participants

Participants were 126 children aged 55-to-77 months (4;7-to-6;4, *M* = 66.72 months; *SD* = 4.72; 113 participants were between 59 and 73 months old). Parental informed consent was required for the children to participate to the experiment. Children were randomly assigned to one of four experimental conditions^1^. Forty children were tested in the Standard condition (18 males; *M* = 66.1 months; *SD* = 5.6; range: 55–75 months), 29 children in the Standard + Verbalization condition (15 males; *M* = 68 months; *SD* = 4.0; range: 60–76 months), 28 children in the A:B-first + No Verbalization condition (14 males; *M* = 65.75 months; *SD* = 2.9; range: 62–73 months), and 29 children in the A:B-first + Verbalization condition 17 males; *M* = 67.4 months; *SD* = 5.0; range: 59–77 months).

#### Materials

**FIGURE 1:**
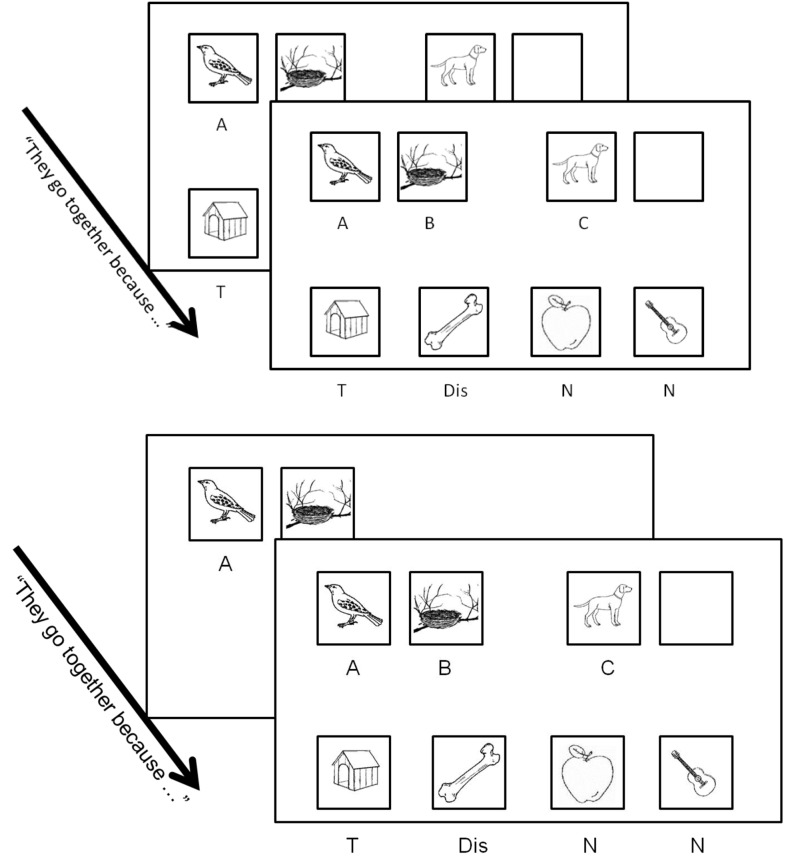
**Design of the Standard/Verbalization (**upper**) and A:B-first/Verbalization conditions (**lower**), (Experiment 1)**.

The same set of analogies was used in all four conditions. It consisted of a set of 14 trials of an A:B::C:? task with two training trials, followed by 12 experimental trials. Most of these analogies came directly from or were adapted from Thibaut et al. (2010a) and were constructed around relations familiar to children (e.g., “is part of,” “lives in,” etc., see Materials, below). Each trial consisted of seven black-and-white drawings (240 × 240 pixels). These were the A, B, and C items, the relational Target (T), a Related-to-C Distractor (Dis), and two Unrelated Distractors (Un) (see **Figure 1**). In the Standard and Standard + Verbalization conditions, all stimuli were presented together at the beginning of the trial. The A, B, and C pictures were presented in a row at the top of the computer screen along with an empty black square where the answer would go. The four possible answers were presented in a row at the bottom of the screen. In the A:B-first + Verbalization condition, the A:B pair was displayed first alone on the screen and the other items were not shown until the participant had verbalized (i.e., spoken aloud) a relation holding between A and B. In the A:B-first + No Verbalization condition, A and B were presented first and participants had to confirm they had studied them. Thereafter, the entire set all items were displayed as in the Standard condition.

For the sake of representativeness, we included the same number of analogies based on weakly semantically associated pairs (called weak analogies, see Thibaut et al., 2010b) and based on strongly semantically associated pairs (called strong analogies). Thibaut et al. (2010b) showed that analogies built around weakly associated pairs (e.g., shirt:suitcase::toy car:box, in which *shirt:suitcase* and toy car:box are weakly associated pairs) were more difficult than analogies built around strongly associated pairs (train:railway track::boat:sea) *even though* children understood the semantic relations between each pair (see Appendix 1 in the Supplementary Materials for the complete list of items).

All trials were presented on a 17-inch élo 1715L touch screen using the E-prime^®^ software. Answer accuracy was recorded during the task.

#### Procedure

The experiment took place in a quiet room at school and children were tested individually. Participants’ knowledge of each stimulus was tested in order to ensure that any incorrect answers in the analogies were not due to a failure to identify a particular item. Each stimulus was introduced separately and the experimenter asked for its name. When children could not name an item, they were asked about its function or where they might find it. When children failed to recognize an item, its name and a short description of it were provided. Before children received the specific instructions for their experimental condition, the experimenter introduced the experiment as a game in which children would see pictures and would have to find “things that went together.” The experimenter also said “we are interested in what *you* think by your answer (emphasis on “you”).”

In the *Standard condition*, the seven images were all shown on the screen at the same time (i.e., A, B, C, and the four possible solution items). Participants then received the following instructions: “Do you see these two pictures [A and B]? They go well together. You first have to find out why they go together. Can you see why they go together? Now, you can see there is another picture here that is alone [C]. When you’ve found out why these two [experimenter pointing toward A and B] go together, you have to find the picture in the bottom [experimenter pointing to the solution set] that goes with this one [experimenter pointing toward C] in the same way as these two [pointing toward A and B] go together. Can you find the one that goes with this one [pointing to C] in the same way as these two [pointing toward A and B] go together?” Children were asked to select an answer from the solution set and “when you find one image, you touch it, and it will climb and go next to this one [pointing to C]. They were then asked to justify their answer by giving the relation that linked A to B, and C to the selected answer. In the two training trials, the experimenter gave feedback for both the correct and the incorrect answers. For correct answers, he/she repeated the reason why correct answers were correct and repeated the instructions. For the incorrect answers, he/she explained what the correct answer was and why, again repeating the instructions. In the following 12 test trials, the experimenter provided no further instructions or feedback. After each analogy, children were asked to explain why A and B, and C and D “went well together.” The experimenter recorded all the stimuli that were chosen and all the justifications.

In the *Standard + Verbalization* condition, children saw all the stimuli at once and were given the same instructions as in the Standard condition. However, they were explicitly asked to verbalize (i.e., report out loud) the A:B relation at the beginning of the trial (“You see these two [pointing to A and B]. Start by telling me why they go well together”). Then the experimenter went on as in the Standard condition. Thus, even though the experimenter first mentioned the A–B pair in the Standard condition, there was no explicit request to verbalize anything. By contrast, in the Standard+Verbalization condition, the experimenter asked the children to first verbalize the relation between A and B. In the *A:B-first + Verbalization* condition, for the two training trials, the experimenter first displayed the A:B pair prior to displaying the five remaining pictures (i.e., C and the four answer options) on the screen. The experimenter said that the other stimuli would be shown later and asked why “the two stimuli go well together” [pointing to A and B]. The other stimuli (C and the four answer options) were shown only *after* participants had verbalized the A–B relation. The experimenter then provided the children with feedback, explained the answer and introduced the second training trial, following the same procedure as for the first training trial. Then the 12 experimental trials were shown (A and B first, verbalization given, followed by the five remaining stimuli) with no feedback. Finally, in the *A:B-first condition + No Verbalization*, in the two training trials, the children were shown only the A:B pair and were told they could study these two stimuli as long as they wished. Once they had told the experimenter they had studied the two pictures, they were shown the five remaining stimuli making up the problem. The training trials went on, as in the previous conditions (pointing, request to explain the relevant relation, etc.). In the experimental trials, the same procedure was followed: A and B were displayed until the children told the experimenter they had studied them. Then, the five remaining stimuli were displayed. No feedback on answer correctness was provided. Encouragement was provided during the task in order to keep children’s motivation as high as possible. After the experimental trials, the experimenter assessed participants’ knowledge of the relations between A and B, and C and T. Indeed, since the main purpose of the present experiments was to study the role of the focus of attention, we wanted to avoid failures resulting from children’ being unaware of the relation holding between the items in a pair. We followed Thibaut et al.’s (2010a) procedure (p. 572). Children were shown the A–B pairs, one by one, and were asked to explain why the two pictures comprising each pair went well together. The same was true for the C–T pairs. Trials in which children could not explain the relation between A and B or between C and the Target were not included in the data set.

### Results and Discussion

Overall, fewer than 2% of the stimuli were not recognized during the children’s knowledge-assessment phase. Forty-four trials out of 1386 were excluded from subsequent analysis because the relation between A and B or C and T(arget) was unknown to the participants.

A two-way ANCOVA with AB-First (Standard, A:B-first) and Verbalization (No Verbalization, Verbalization) as between-subject factors was run on the performance scores of children (i.e., the number of correct relational choices), with age as a covariate (because the age range was close to 2 years). It revealed a significant effect of A:B-first [*F*(1,121) = 4.61; *p* = 0.034; η^2^= 0.037; means are 60%, and 66% correct for the Standard, and A:B-first respectively]. The Verbalization factor was also significant [*F*(1,121) = 4.14; *p* = 0.044; η^2^= 0.033, 60% correct in the No Verbalization condition, and 65% in the Verbalization condition]. The interaction between these two factors was not significant [*F*(1,121) = 0.57; *p* = 0.45; η^2^= 0.005]. The Age (covariate) was not significant [*F*(1,121) = 0.44; *p* = 0.51; η^2^= 0.004]. Note that, as in previous experiments (e.g., Thibaut et al., 2010a) most of the errors (more than 80%) involved choosing the semantic distractor. This is consistent with Thibaut and French (2016) who have shown, by means of eye-tracking, that children spend a considerable amount of time comparing the analogical target to the semantic distractor and each of these items with C. This suggests that participants processed the semantic distractor and the analogical target items before making a decision.

The unbalanced attentional focus hypothesis posits that children’s failures in these analogy tasks could be due to their over-attention to the main goal of the task (C:? subgoal) at the expense of an analysis of the A:B pair (see Thibaut and French, 2016). Our results confirmed this hypothesis. The significant effect of the A:B-first factor shows that the prior viewing of the A:B pair contributes to the inclusion of the relation between A and B into the problem. It allowed children to process this relation, thereby making it more available (i.e., activated), when the remaining stimuli were introduced. Within this task format, inhibiting the C-?-goal was less of a problem: participants focused on the A:B pair and integrated it with the rest of the problem. This result suggests that the way the task is paced influences children’s integration of the different parts of the task.

Similarly, verbalizing the A:B relation also significantly improved children’s performance. In keeping with the unbalanced attentional focus perspective, children’s naming of the A:B relation contributes to focusing their attention toward this pair, thereby integrating it with the other information provided (see Introduction).

## Experiment 2

In Experiment 1, in line with the framework of the unbalanced attentional focus hypothesis, we showed that helping children to organize their search in order to build and integrate various sources of information was important for analogy making. It showed that both A:B-first and Verbalization contributed to reinforcing the A:B pair by appropriately segmenting the task, and focusing children’s attention on the relation between the two pictures. In Experiment 2, we pursued this line of reasoning. We showed that inducing children to encode an irrelevant A:B relation had a disruptive influence on their performance by contrasting two groups of analogies. In one condition, the A:B pair was constructed in such a way that there were two relations that could be applied to the items of the A:B pair. The first relation was a semantic relation such as “lives in.” This relation was the one that made sense of the entire analogy, the second relation was always the “same color” relation (A and B were of the same color). We hypothesized that the same color relation would be the first to be noticed because it is perceptually grounded (see Rattermann and Gentner, 1998, for a discussion). These two types of analogies were used in two experimental condition, first, the A:B-first + Verbalization condition from Experiment 1 and, second, a very slightly modified version of the Standard condition (hereafter, the Standard-3sec, see Procedure). The key hypothesis in the present experiment was that the irrelevant dimension (i.e., color) would produce more interference in the A:B-first + Verbalization condition than in the Standard condition. Indeed, as suggested by the unbalanced attentional focus hypothesis and by Experiment 1, if children in the Standard-3sec condition (i.e., no verbalization) organize their search around C (see Thibaut and French, 2016), they should be less influenced by the irrelevant relation (color) in the A:B pair. By contrast, in the A:B-first + Verbalization condition children would have difficulty switching from their initial representation of the relation (Fabricius, 1988; Zelazo et al., 2003; Garon et al., 2008; Blaye and Chevalier, 2011) and, in addition, finding a new relation between the A:B pair once the first one is found to be irrelevant has costs that should affect children’s performance.

We also introduced a third type of analogy in which the “same color” relation was, in fact, relevant in finding a solution. This was done to ensure that the same color relation remained a possible solution throughout the task and, thus, would not simply be ignored after a small number of trials.

### Methods

#### Participants

Participants in this experiment were 46 62-to 84-month-old children (28 males; *M* = 70.6; *SD* = 5.9). Twenty-two children participated in the AB-first + Verbalization condition (10 males; *M* = 69.4 months; *SD* = 3.7; range: 63–76 months) and 24 in the Standard-3sec condition with no verbalization (18 males; *M* = 71.7; *SD* = 7.4; range: 62–84). Parental informed consent was required for them to participate in the experiment.

#### Materials

**FIGURE 2:**
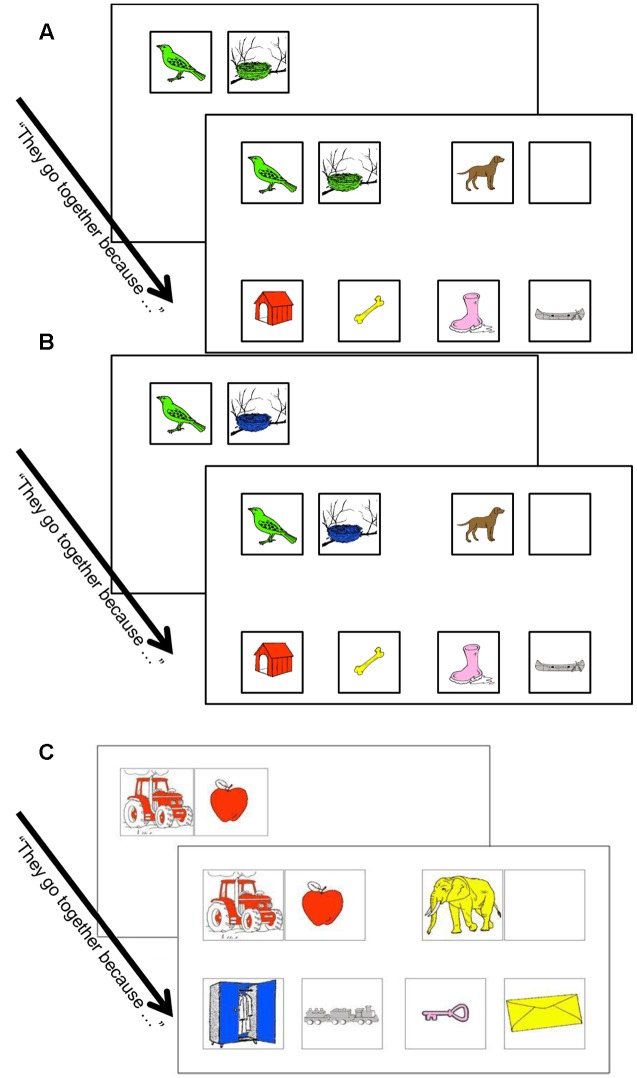
**Examples of AB-Color-Semantic-match (A)** of AB-Semantic-match trials **(B)** and color trials **(C)** in Experiment 2. In the color trials, the “non-solution” stimuli were not semantically related to C. Examples coming from the AB-first + Verbalization condition.

The task consisted of 13 A:B::C:? problems, i.e., 2 training problems and 11 experimental problems. As in the previous experiment, each trial consisted of seven line drawings (240 × 240 pixels) for the A, B, C items, the relational Target (T), a Related-to-C Distractor (Dis) and two Unrelated Distractors (Un) (see **Figure 2**). In contrast to Experiment 1, where no colors were used, in this experiment each drawing was filled with a single color (red, blue, yellow, green, rose, red, brown, or gray).

The 11 experimental trials were divided into three categories. *First*, there were four AB-Semantic-match trials in which A and B were linked by a semantic relation (e.g., “has a” for the items “man” and “nose”), and C:Relational-Target had the same semantic relation which was, thus, the analogical relation (e.g., C was “moose” and the Relational-Target was “muzzle”). In this condition, there was no other obvious relation between A and B (A and B were of different colors). These trials were equivalent to those in the first experiment.

*Second*, four AB-Color-Semantic-match trials, in which A and B were related by both a semantic relation, as above, and, in addition, they were related by an “identical-color” relation, whereas, for the C: Relational-Target pair, only the semantic relation was relevant to solve the analogy. In other words, these trials were designed in such a way that, when considering the C item and the solution set, only the semantic relation made analogical sense (i.e., there was no possible “same color” solution). Thus, the only difference between AB-Semantic-match and AB-Color-Semantic-match trials was the differing use of color. The AB-Color-Semantic-match trials and the AB-Semantic-match trials that were seen by half of the participants were transformed into AB-Semantic-match trials and AB-Color-Semantic-match trials, respectively, for the other half of the participants. In both of these conditions, there were only semantic distractors and no perceptual lures. Examples of AB-Semantic-match and AB-Color-Semantic-match trials are shown in **Figure 2**.

*Third*, there were three Color trials in which the analogical relation was “same color as.” A and B were of the same color and participants had to find an item that had the same color as C. These trials were constructed in such a way that no obvious plausible semantic relation could be found. These trials ensured that “same color as” remained a possible relational solution throughout the task. In order to ensure that children would not simply ignore the “same-color” relation, we interspersed one of the three Color trials between two trials of the other types (i.e., AB-Semantic-match and/or AB-Color-Semantic-match trials). Note that in all the stimuli across conditions, we colored the stimuli uniformly with one color that often differed from the real color of the object. This was done to enhance color saliency and make it a dimension of the stimuli. In this way, our stimuli differed from their real world counterparts in which colors often have different shades (i.e., are not uniformly distributed on the object) (see Appendix 2 in the Supplementary Materials for the complete list of items).

As in Experiment 1, the two association strength (“Strong” or “Weak”) were balanced across conditions. We used these two types of trials for the sake of representativeness (see Thibaut et al., 2010a, for a discussion of this distinction). 50% of the AB-Semantic-match and AB-Color-Semantic-match trials were composed of weakly associated pairs, and 50% of strongly associated pairs as defined in Experiment 1.

We also constructed two versions of the stimuli, which differed by the Related-to-C distractors that were used. For example, in one version, the related-to-C distractor was “*whiskers*” (C being “*cat*”) and in the other version, it was “*dog.*” The mean association strength between C items and the two sets of distractors was not significantly different (two-tailed Student’s *t*-test, *p* > 0.05).

The task was presented on a 17′′ élo 1715L touch screen with the mean of an E-prime^®^ software. Answer accuracy was recorded during the task.

#### Procedure

The same procedure as in Experiment 1 was used to assess children’s knowledge of the stimuli. In the AB-first + Verbalization condition, the A:B pair was displayed, and, once the A:B relation had been verbally provided by the child, the remaining items making up the problem were displayed. In the Standard-3sec condition (i.e., with no verbalization of the A:B relation), participants were shown A, B and C for three seconds. (This is a slight modification with respect to the Standard condition procedure in Experiment 1 in which all items, including the solution set were presented from the outset. We wanted to be as close as possible to the AB-first + Verbalization condition but with no *explicit* request to verbalize the AB relation.) The training phase instructions and feedback were the same as in the Standard condition (i.e., with no verbalization) in Experiment 1. Participants received no instructions and no feedback during the test trials. As in Experiment 1, the session ended with the assessment of participant’s knowledge of the relations composing the analogies.

### Results and Discussion

We removed one participant who answered exclusively in terms of color relations for all trials from the data set. Only 1% of the items presented in the first phase were not spontaneously labeled or described accurately. Six trials out of 517 were not analyzed due to a lack of knowledge of one of the semantic relations between items.

**FIGURE 3:**
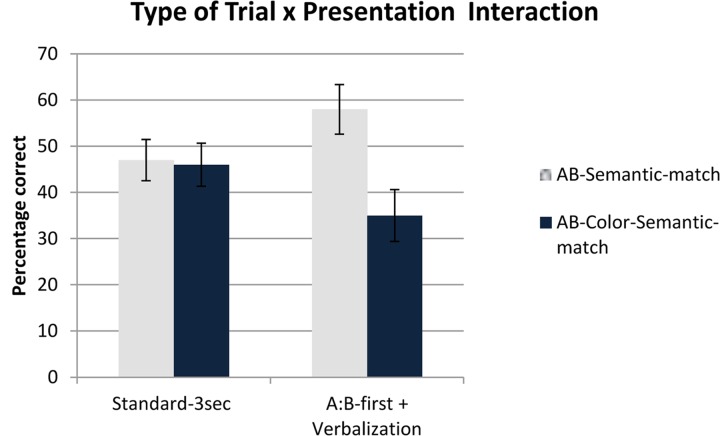
**Type of Trial × Presentation interaction in Experiment 2 showing a significant difference between AB-Semantic-match trials and AB-Color-Semantic-match trials in the AB-first + Verbalization and no difference in the Standard-3sec condition**.

A two-way mixed ANCOVA was performed on the percentage of correct trials for AB-Semantic-match, and AB-Color-Semantic-match trials, with Presentation (AB-first + Verbalization, Standard-3sec) as a between-participants factor and Type of Trial (AB-Semantic-match, AB-Color-Semantic-match) as a within-participants factor. Age was introduced as a covariate. This analysis revealed no significant main effect of Type of Trial (*p* > 0.10) and no significant effect of Presentation (*p* > 0.10). There was a positive effect of the covariate factor Age [*F*(1,43) = 5.78, *p* = 0.02, η^2^= 0.12]. Following our unbalanced attentional focus hypothesis, the most interesting result was the significant interaction between Type of Trial and Presentation [*F*(1,43) = 5.63, *p* = 0.021, η^2^= 0.12]. A Tukey HSD *post hoc* analysis showed that performance on AB-Semantic-match trials was better than on the AB-Color-Semantic-match trials in the AB-first + Verbalization condition (58% vs. 35% correct, respectively; *p* = 0.010). However, crucially, these two conditions did not differ in the Standard-3sec condition (47% vs. 46% correct respectively; Tukey HSD, *p* = 1). In order to better understand what the role of the color relation was, we performed two separate comparisons of the AB-First + Verbalization and Standard-3sec conditions, one for the AB-Semantic trials, the other for the AB-Color-Semantic trials. The two contrast analyses revealed no significant effect (*p* > 0.10). This is because the data for both the AB-Semantic match and AB-Color-Semantic match in the Standard-3sec condition fell in between the data for these two conditions in the AB-First condition. This suggests that the AB-first had both positive (increased performance when the irrelevant color relation was absent) and negative influence (decreased performance when the color relation was present). Finally, performance on color-relevant trials (i.e., when color was the relevant dimension for solving the problem) was quite good (85 and 86% correct, respectively) and did not differ in the two conditions (AB-Semantic- and AB-Color-Semantic) [*t*(44) = 0.6, *p* > 0.5]. This confirmed that the color relation remained activated and available during the entire experiment (**Figure 3**).

These results confirm the unbalanced attentional focus hypothesis, according to which the AB-first + Verbalization condition would help children to focus and assimilate the relational information in the A:B pair. This means that in the AB-Color-Semantic-match condition, children first selected the same-color relation for the A:B pair. Given that this relation was irrelevant for solving the analogy problem, it interfered with their search for the correct analogical solution. This led to more errors in this condition than in the AB-Semantic-match condition in which the same-color relation was absent in the A–B pair.

In contrast, as predicted by the same hypothesis, in the Standard-3sec condition, there was no difference between the AB-Color-Semantic-match and the AB-Semantic-match conditions. In other words, in this condition performance was unaffected whether or not there was the “same color” relation between A and B. Hence, when children were not asked to focus on the A:B pair and the relation between A and B, the presence of the color relation did not influence their performance. We believe that focusing on C first and rapidly distributing their attention to both the solution set and to B, as suggested by Thibaut and French (2016), might have led to an early activation of the semantic relations holding between C and the relational Target and between C and the semantic distractor. In this case, the irrelevant same-color relation would have less influence on the search for a solution. Thibaut and French, 2016 also showed that children turned their attention to B quite early, but to A somewhat later. This early focus on C, the Target and the Semantic Distractor would cause these three stimuli to become active, so that the children are less influenced by the “same color” relation when the A–B pair is focused on. Hence, the comparison between the two Presentation conditions suggests that the important factor is “how the search is organized” rather than the presence of an obvious-but-irrelevant relation.

## General Discussion

In our data, we found evidence for the effect of search organization in solving analogy tasks by children, an effect that has been largely overlooked in the literature. Indeed, according to most studies, knowledge accretion and/or difficulties inhibiting irrelevant interpretations or distractors would be sufficient to explain children’s difficulties in analogical reasoning tasks. Here, we tested what we have called the *unbalanced attentional focus hypothesis*, according to which children’s failures might also result from difficulties in focusing their attention on both the base and the target pairs. We tested this hypothesis (i) by manipulating the order in which the information was made available (i.e., prior presentation of the A:B pair) and (ii) by requiring the children to verbalize relational information between the A:B items of the problem they were attempting to solve. There were two key results. First, Experiment 1 revealed main effects of both Verbalization and Prior presentation of the A:B pair. Second, Experiment 2 showed that the presence of a salient, but irrelevant, relation between A and B (same color) had a detrimental effect only when children were explicitly incited to focus on the A:B pair (A:B-first + Verbalization condition). Further, when there was no additional, induced emphasis to focus on the A:B pair (i.e., in the Standard condition), the salient-but-irrelevant “same color” relation in the A:B pair had no deleterious influence on performance. Together, these results demonstrate that the way the task is temporally segmented (i.e., organized) influences children’s analogical problem-solving performance.

### Analogical Reasoning Development, Information Search and Integration, and Executive Functions

Searching for the solution to an analogy problem requires the integration of a multitude of information, which requires adequate focus on the information available. Our results show that the organization of the task plays a crucial role in performance. In their eye-tracking study, Thibaut and French (2016) showed that young children tended to spontaneously focus less on the A:B pair than adults and organized their search around C. They speculated that this lack of focus on C contributed to children’s poorer performance compared to adults. We claim that in the Standard condition, the explicit main goal of the analogical task (i.e., “finding the stimulus that goes with C”) is difficult to inhibit, thus preventing the child to focus on the A:B pair. The factors we manipulated contributed to enhancing the “encode the A:B pair” subgoal.

Experiment 1 revealed that the effect of showing the A–B pair first and verbalizing the relation between A and B produced the best results obtained when they were combined (i.e., the A:B first + Verbalization condition). In Experiment 2, there was a significant difference between the AB-Color-Semantic-match condition (i.e., same color) and the AB-Semantic-match condition (i.e., different colors) only in the A:B first + Verbalization condition, when explicit encoding of the color relation between A and B was induced. In this case, children subsequently had to inhibit their initial (and irrelevant) color-based representation of A:B and flexibly find a novel relation between A and B that was consistent with the relation available in the target pair. By contrast, in the Standard-3sec condition, the irrelevant same-color dimension of the A:B pair had no effect on the performance (i.e., there was no significant difference between the AB-Color-Semantic-match and the AB-Semantic-match conditions). This difference between the Standard-3sec and the A:B-first + Verbalization conditions is compatible with our unbalanced attentional focus hypothesis, and, more broadly, with an executive-function framework. In the Standard-3sec condition the irrelevant same-color dimension in the A:B pair had no effect on performance because the presence of C at the beginning of the trial, combined with the explicit “C:? goal,” led children to start with the strategy described by Thibaut and French (2016, see above). Activating the “find what goes with C” instruction (see above) interfered with the secondary subgoal of “finding the A:B pair relation.” Consequently, the irrelevant A:B relation (“same color”) interfered less, which resulted in no significant difference between the AB-Color-Semantic-match and the AB-Semantic-match conditions.

### The Unbalanced Attentional Focus Hypothesis and Executive Functions

The knowledge accretion view cannot account for the present data in a straightforward manner, since within each experiment, the same set of analogies was used across conditions that differed only in terms of stimulus display timing and verbalization. Also, the analyses were performed on analogies for which children could explain the relation for both base and target pairs in the post-experiment assessment. The mainstream view of the “EF” explanation of the development of analogical reasoning usually refers to the necessity of inhibiting irrelevant information, such as semantically and/or perceptually related distractors (e.g., “*bone*” in the “*bird:nest::dog:?”* analogy; see Richland et al., 2006; Thibaut et al., 2010b) or to the number of relations to process in working memory (e.g., Halford et al., 1998). Our unbalanced attentional focus hypothesis (and Thibaut and French, 2016) suggests that other factors need to be added to the EF explanation, factors that are associated with the temporal organization of the task that will allow re-representation of a pair of stimuli. Again, it is important to emphasize that in the analogy literature the concept of inhibition has not previously been related to the temporal organization of the task by children. The necessity of taking this temporal organization into account is the central point of the present contribution.

The present framework also sheds new light on the role of language in analogy making. In their review of language influences on cognition, Wolff and Holmes (2011) propose that language impacts thinking in various ways, what the authors call “before language,” “with language,” and “after language.” In previous studies, highlighting concepts “with language” had a positive effect when the experimenter gave a name (vs. no name) to the objects or the relations in the pairs at the start of a trial (e.g., Loewenstein and Gentner, 2005). In this case, the effects of naming can be explained by the activation of the representation of the stimuli dimensions associated with the name, what Gentner (2010) calls *reification*.

Our data provide another instance of the “with language” influence that has been identified by Wolff and Holmes (2011). Here, asking children to *name the relation* between A and B, directed their attention to the A:B subgoal, i.e., to the A and B items, thereby explicitly encouraging participants to compare them. Language was used to highlight a *specific part of the task*, a part that we hypothesized did not receive sufficient attention at the beginning of the trial. Here, language contributes to help children to organize the task. However, it’s not attention toward A–B *per se* that elicited better results, but most likely deeper processing of the pair. Indeed, if children did not look at the A–B pair, they would be unable to process it and find the relation holding between A and B. Note that in their comparison of correct answers and errors, Thibaut and French (2016) showed that when a problem was answered erroneously, there were *fewer* gazes to A and B at the beginning of the trial than when a correct answer was given. In most cases, errors involved the selection of the distractor that was semantically related to C. This is likely to occur if one does not process the A:B pair, or processes it inadequately.

Thus, verbalization contributed to children’s processing the A:B pair, which produced a significant positive improvement in performance in Experiment 1. It also contributed to disrupting performance in the AB-Color-Semantic-match condition in Experiment 2. As in our experiments, Kray et al. (2008) also found significant effects of verbalization. When forced to verbalize information relevant to the ongoing task, children showed better performance on the task, whereas irrelevant verbalization interfered with the task. In our case, language played the same focusing role and helped children to focus on an *a priori* neglected component of the task – namely, the base pair. Similarly, in a color selection task, Müller et al. (2004), showed that performance was facilitated when the experimenter pointed to relevant information (a card of a given color was associated with an M&M). This manipulation was interpreted as directing attention toward the relevant information. This could be seen as analogous to our AB-first condition or when participants were asked to verbalize the A:B pair. These manipulations directed attention toward the A:B pair and facilitated its encoding. By contrast, in Experiment 2, the detrimental effect of the obvious-but-irrelevant information (the color relation in the A:B first + verbalization condition) is analogous to detrimental effects associated with irrelevant verbalizations (e.g., Gruber and Goschke, 2004). Once participants have been requested to focus their attention toward the A:B pair, their verbalization of the relation might also contribute to making it cognitively more salient, and thus more difficult to inhibit when it is irrelevant, as in Experiment 2. In our experiments, we did not control for participants’ linguistic competence (e.g., vocabulary). It was assumed that their language level was essentially equivalent across conditions. One further step would be to control for children’s linguistic level and to include this factor in the model in order to determine, for example, whether better linguistic levels would positively correlate with performance. It might also be that the effect of language could be smaller for children with lower linguistic competence (once age differences are controlled for).

The ability to temporarily disengage from the main goal of a task and to focus on other information that is crucial to the completion of the primary goal has been shown in recent years to be central to problem solving abilities and has been extensively studied in the cognitive flexibility literature. Compared to the standard Dimensional Change Card Sort (DCCS), the Advanced DCCS is a cued task switching paradigm and introduces a mixed block in which shape and color alternate unpredictably, each dimension being the relevant classification criterion depending on the nature of a visual cue. Chevalier et al. (2010) have shown that children first focus on the target information (color or shape) before they fixate the cue that tells them which dimension is relevant, whereas adults do the opposite. Thus, in both Chevalier et al. (2010) and Thibaut and French (2016), children’s errors are (at least in part) due to their inability to shift away from the main goal of the task and to integrate information (the A:B pair in our case, the cue in Chevalier et al., 2010) that is crucial for correct task completion.

Thus, in addition to knowledge accretion and inhibition of irrelevant distractors, our results show that the way children inhibit the main goal of the task and/or consider all the information available is important and contributes to the explanation of children’s failures in analogical reasoning tasks.

### Generality of the Findings

We believe that the present results can be generalized to other analogy paradigms, such as scene analogies (Richland et al., 2006). Richland et al. (2006) reported poor results for the 3- to 4-year-old group (65% correct responses) or even for the 6- to 8-year-old group (80% correct), in the easiest “no distractor” condition and much worse performance when a distractor was present. We believe that our *unbalanced attentional focus hypothesis* also applies here. The instructions are analogous to those in the A:B::C:D task. Both scenes are mentioned by the experimenter. The main goal, i.e., “Which one is in the same part of the pattern in the bottom picture? [The experimenter pointed to each object as it was described]” (p. 256), refers to a choice between stimuli in the bottom target scene. Performing the task requires inhibition of the target scene and a shift to the “source scene” in order to identify the relation holding between the stimuli and the role played by each stimulus. One then comes back to the target scene in order to identify the corresponding stimulus. Thus, children’s difficulties in the scene task might also be due, at least in part, to difficulties involving shifting their attention away from the target scene (i.e., temporarily “defocusing” their attention to the target scene and “refocusing” it on the source scene).

The same reasoning might also apply in an analogy problem-solving task. One has to temporarily inhibit the main task and analyze the source problem. In general, comparing the source pair and the target pair (or target scene or target problem) requires disengaging one’s focus from the main goal. A failure to do so arguably results in poorer encoding of the source, poorer identification of the relation holding between the source stimuli, poorer alignment of the roles, etc.

## Conclusion

The results of the present study support the view of the development of analogical reasoning capacities as being constrained by both executive-function maturation and strategy learning (i.e., using verbal labels to sequentialize the task), both of which are involved in producing an adequate strategy when solving problems of the A:B::C:? type. The present study shows how these planning difficulties can be decreased by modifying the procedure used in the task — namely, by inducing children to focus on the relation between A and B and to verbalize the relational information of the A:B pair. However, inducing explicit focus on the A:B pair may raise other problems if the information found is not relevant to solving the problem. In this case, children must be flexible in their representation of the source and target domains and in the strategy used to find the solution. Most previous models have taken into account EFs constraints separately, whereas the present work attempts to show the importance of integrating working memory, inhibition and cognitive flexibility.

## Ethics Statement

There is a written and official agreement between our laboratory, the University of Burgundy, and the Inspection Académique of the Côte d’Or in charge of the schools in our area. Written consent is obtained from parents.

## Author Contributions

YG: designed Experiment 1, collected and analyzed the corresponding data. Participated in the writing process. RF: design, participated in the writing process. J-PT: design of Experiments 1 and 2, data analysis and writing process.

## Conflict of Interest Statement

The authors declare that the research was conducted in the absence of any commercial or financial relationships that could be construed as a potential conflict of interest.

## Footnotes

1 In fact, not all participants were randomly assigned to one of the four conditions. Due to a minor misunderstanding at the beginning of the experiment, 10 participants were seen in the standard condition and none in the other conditions. Afterward, this was corrected and participants were assigned to each of the four conditions randomly. To be sure that this error had no influence on the data, we compared these first 10 participants data with the remaining participants’ data in the same condition and found no difference in the mean, and, thus, kept all the data.
