# Supplementary material for: Children’s Failure in Analogical Reasoning Tasks: A Problem of Focus of Attention and Information Integration?
Source: Front Psychol. 2017 May 23;8:707. doi: 10.3389/fpsyg.2017.00707 (PMC5440766; doi:10.3389/fpsyg.2017.00707)
Supplement: Supplementary file 1 [file Data_Sheet_1.docx]

Appendix A: List of the test trials for Experiment 1.

| Weak Associations | | | | Strong Associations | | | |
| --- | --- | --- | --- | --- | --- | --- | --- |
| Analogy problem | Target | Distractors | | Analogy problem | Target | Distractors | |
|  |  | Related | Unrelated |  |  | Related | Unrelated |
| shirt: suitcase:: toy car | box | fuel pump | tree, monkey | train: rail:: boat | sea | crab | leave, banana |
| child: bed:: cat | pillow | whiskers | guitar, strawberry | mitten: hand:: shoe | foot | footprint | trumpet, croissant |
| pig: trough:: man | dish | watch | plane, tractor | lamp: outlet:: remote control | battery | stereo system | bucket, boot |
| man: nose:: moose | muzzle | owl | pen, bell | bird: nest:: dog | doghouse | bone | chair, glasses |
| glass: dresser:: ring | case | watch | rope, skittle | spider: web:: bee | beehive | flower | hat, kayak |
| pineapple: bottle:: orange | carafe | strawberry | feather, bench | door lock: key:: bottle | bottle opener | glass of wine | hat, kayak |

Appendix B: List of the test trials for Experiment 2

| Weak Associations | | | | Strong Associations | | | |
| --- | --- | --- | --- | --- | --- | --- | --- |
| Analogy problem | Target | Distractors | | Analogy problem | Target | Distractors | |
|  |  | Related | Unrelated |  |  | Related | Unrelated |
| shirt: suitcase:: toy car | box | fuel pump | carrot, leaf | train: rail:: boat | sea | crab | goat, pen |
|  |  | motorcycle |  |  |  | fish |  |
| child: bed:: cat | pillow | whiskers | bell, trumpet | mitten: hand:: shoe | foot | footprint | wolf, pan |
|  |  | dog |  |  |  | shoelace |  |
| pig: trough:: man | dish | watch | bee, cage | lamp: outlet:: remote control | battery | switch | horse, robe |
|  |  | pullover |  |  |  | stereo system |  |
| glass: dresser:: ring | case | watch | giraffe, grass | bird: nest:: dog | doghouse | bone | boot, kayak |
|  |  | necklace |  |  |  | puppy |  |
